# The Mental Health Quality of Life Questionnaire (MHQoL): development and first psychometric evaluation of a new measure to assess quality of life in people with mental health problems

**DOI:** 10.1007/s11136-021-02935-w

**Published:** 2021-07-09

**Authors:** F. C. W. van Krugten, J. J. V. Busschbach, M. M. Versteegh, L. Hakkaart-van Roijen, W. B. F. Brouwer

**Affiliations:** 1 Erasmus School of Health Policy & Management, Erasmus University Rotterdam, PO Box 1738, 3000 Rotterdam, The Netherlands; 2 Department of Psychiatry, Section Medical Psychology, Erasmus Medical Center, Rotterdam, The Netherlands; 3 Institute for Medical Technology Assessment, Erasmus University Rotterdam, Rotterdam, The Netherlands

**Keywords:** MHQoL, Quality of life, Mental health, Preference based measures, Development, Psychometric evaluation

## Abstract

**Purpose:**

The purpose of this study was to develop and psychometrically evaluate a new quality of life measure for use in people with mental health problems—the Mental Health Quality of Life questionnaire (MHQoL).

**Methods:**

The MHQoL dimensions were based on prior research by Connell and colleagues, highlighting the seven most important quality of life dimensions in the context of mental health. Items were generated following a systematic review we performed and through inviting expert opinion. A focus group and an online qualitative study (*N* = 120) were carried out to assess the face and content validity of the MHQoL. The MHQoL was further tested for its internal consistency, convergent validity, known-group validity and test–retest reliability among mental healthcare service users (N = 479) and members of the general population (*N* = 110).

**Results:**

The MHQoL consists of a descriptive system (MHQoL-7D), including s items covering seven dimensions (self-image, independence, mood, relationships, daily activities, physical health, future) and a visual analogue scale of general psychological well-being (MHQoL-VAS). Internal consistency was high (Cronbach's *∝* = 0.85) and correlations between MHQoL-7D scores and related measures (EQ-5D-5L, MANSA, ICECAP-A, and BSI) supported convergent validity. The intraclass correlation coefficient of the MHQoL-7D sum score for test–retest reliability was 0.85. Known-group validity was supported by the ability to detect significant differences in MHQoL-7D levels between service users and the general population, and between groups with different levels of psychological distress.

**Conclusion:**

The MHQoL demonstrated favourable psychometric properties and showed promise as a simple and effective measure to assess quality of life in people with mental health problems.

**Supplementary Information:**

The online version contains supplementary material available at 10.1007/s11136-021-02935-w.

## Introduction

The concept of quality of life is widely and increasingly used as an important outcome measure in the evaluation of healthcare interventions [1]. Also in the mental health field, it is recognized that while symptom reduction is a desirable treatment outcome, it is also important to assess how recovery translates to the daily life of an individual and their quality of life [2]. Although a consensual definition is lacking, there is general agreement that quality of life is a subjective and multidimensional construct that captures an individual's life satisfaction and overall well-being [3]. In order to accommodate the growing interest in measuring and monitoring the impact of mental health(care) on peoples’ lives, mental healthcare providers in, for example, the Netherlands and the United Kingdom, increasingly include quality of life measures in their routine outcome measurement alongside more clinically oriented measures [4, 5].

Despite the growing interest in assessing quality of life in mental healthcare, it has been questioned whether frequently used quality of life measures, such as the EuroQol five-dimensional (EQ-5D) questionnaire [6] and the 36-item Short-Form Health Survey (SF-36) [7], adequately capture and value the benefits of mental healthcare interventions. Previous studies have indicated that frequently used quality of life measures are, in certain situations, not sufficiently sensitive to the effects of mental health problems on quality of life [8–11]. It has been argued that this may be due to the large focus on physical health of these commonly used quality of life measures, which limits the coverage of the dimensions of quality of life that are valued highly by people with mental health problems [8].

A recent systematic review [12] indicated that the inability of available quality of life measures to adequately capture and value the benefits of mental healthcare interventions might be related to the content validity of those measures. More specifically, it was found that none of the generic (e.g. SF-36 [7]), domain-specific (e.g. Manchester Short Assessment of Quality of Life [13]) or disease-specific (e.g. Schizophrenia Quality of Life Scale [14]) quality of life measures used in people with mental health problems fully cover the dimensions that were found to be important to the quality of life of people with mental health problems [15, 16]. Those findings underline the need for a measure that covers the dimensions considered to be important by people with mental health problems, providing both a descriptive profile and an overall index.

The present paper reports on the development and psychometric evaluation of the Mental Health Quality of Life questionnaire (MHQoL), designed to comprehensively provide information about the quality of life dimensions known to be relevant across and valued highly by people with mental health problems. The conceptual framework was established based on previous work carried out by Connell and colleagues [15, 16]. This work aimed to identify the dimensions of quality of life important to people with mental health problems and has been shown to be an attractive theoretical foundation for the development of quality of life measures for use in the mental health field. Indeed, in the same period in which the MHQoL was developed, Keetharuth and colleagues developed the Recovering Quality of Life (ReQoL) measures [17], which were also based on this framework. In the discussion section of this paper, we will reflect on the differences between the MHQoL and the ReQoL measures.

## Methods

The study consisted of two major phases: (1) development and (2) psychometric evaluation of the Mental Health Quality of Life Questionnaire (MHQoL). The study was reviewed and approved by the Medical Ethical Committee of the Erasmus University Medical Centre Rotterdam, The Netherlands (MEC-2018-142) and digital informed consent was obtained from all participants in the study.

### MHQoL development

**Fig. 1:**
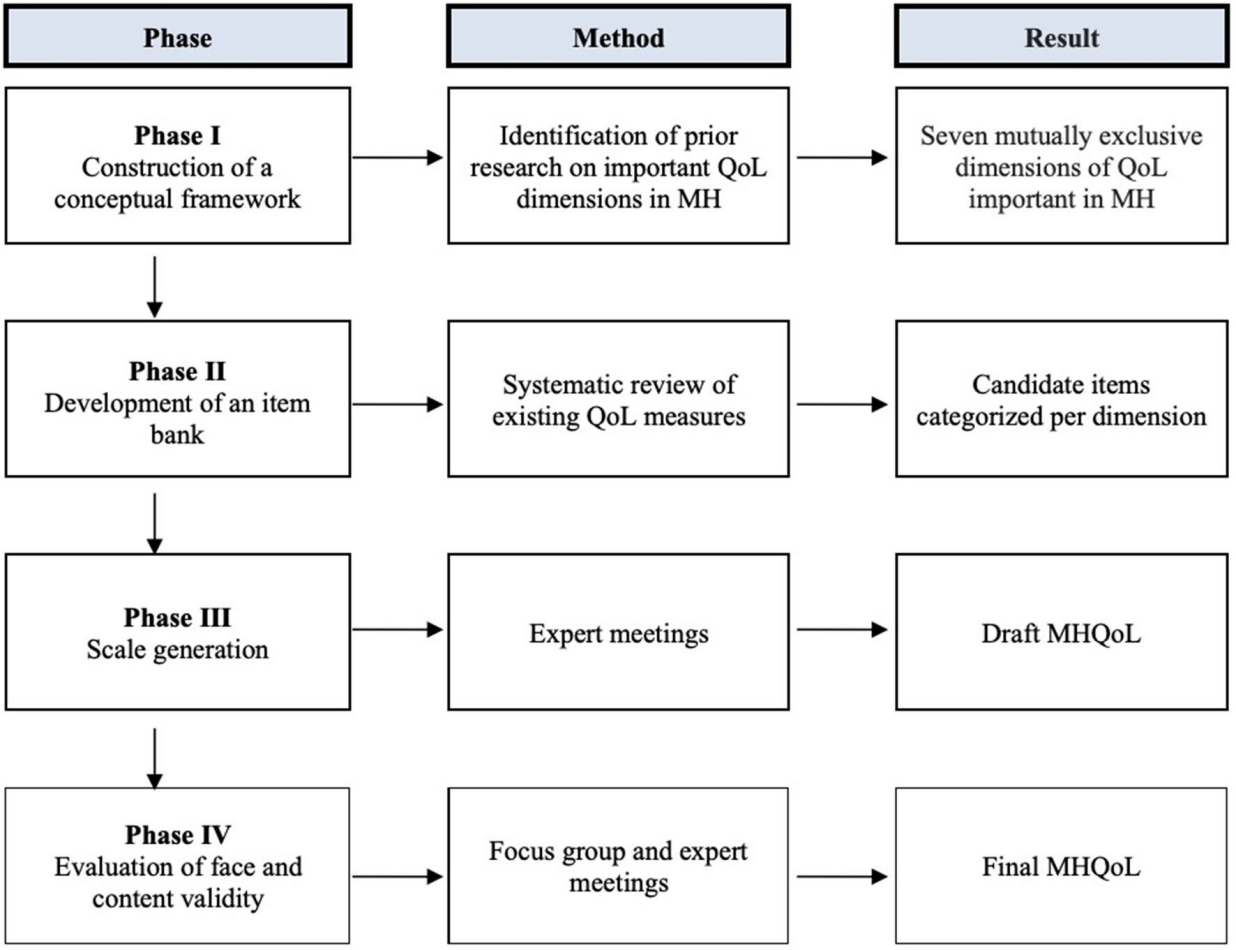
Development stages of the MHQoL. *QoL* Quality of Life, *MH* Mental Health, *MHQoL* Mental Health Quality of Life questionnaire

The first phase of the study, in which the MHQoL was developed, consisted of four stages: (I) construction of a conceptual framework to guide measurement development; (II) development of an item bank to guide item generation; (III) scale generation; and (IV) evaluation of face and content validity. See Fig. 1 for a summary of the phases. The development process was led by a group of researchers (*n* = 6, 5 of whom are co-authors) with relevant expertise in the field of scale development, mental healthcare, or in both.

As a first stage in the development process, a conceptual framework was constructed to serve as a theoretical basis for the resultant measure. The conceptual framework was established based on previous work carried out by Connell et al. [15, 16], highlighting seven dimensions of quality of life most important to people with mental health problems (well-being and ill-being; physical health; autonomy; self-perception; relationships and belonging; activity; hope and hopelessness). The work by Connell and colleagues [15, 16] was selected as the basis for the conceptual framework, given that it specifically aimed to identify the dimensions of quality of life important to people with mental health problems by using a rigorous mixed-methods approach combining a systematic review of qualitative research [15] with complementary interviews [16]. A visual representation of the dimensions of the conceptual framework can be found in the work of Keetharuth et al. [18]. In the second stage of the development process, a bank of candidate items was developed to inform the generation of MHQoL items. The item bank was developed on the basis of a recent systematic review we performed that aimed to identify existing quality of life measures used in people with mental health problems [12]. Through examination of the content of the identified measures (*n* = 35), a total of 272 candidate items were extracted and categorized per dimension of the conceptual framework. In three expert meetings, the item bank was reduced by only retaining the items that best covered the underlying themes of the dimensions of the evaluation framework (see Connell et al. [15] for the underlying themes of the dimensions).

Informed by the reduced bank of candidate items, preliminary scale items were generated for each of the seven dimensions of the conceptual framework in the third stage of the development process. Main requirements in the generation of items were that the resultant measure should be trans-diagnostic in nature and short and easy to complete by the respondent. These principles led to the operationalization of the seven dimensions into seven items (one item per dimension), each with four response options.^1^ In line with measures like the EQ-5D and in order to avoid subjective weighting of health states experienced over longer periods of time, the recall period was set to "today". In 12 expert meetings the generated items were extensively discussed to ensure that all items sufficiently reflected the intended meaning of each of the dimensions. As a result of the discussions, some changes were made to the wording and labels of the items, resulting in the first draft version of the MHQoL.

In the fourth and final stage of the development process, the face and content validity of the draft version of the MHQoL were evaluated in two steps. The first step consisted of a focus group in which six mental healthcare service users were asked to complete the MHQoL, followed by a de-briefing exercise in which they examined the meaning of the individual items, the extent to which the items seem to cover the things that matter in their lives, and the adequacy of the response options. Based on this focus group, minor changes were made to the wording and sequence of the items. In the second stage, a web-based survey was carried out among 120 adult (18 years and older) mental healthcare service users. Participants were randomly drawn from an online panel through the market research company Dynata. Inclusion criteria were: aged 18 years or older and visited any health professional (e.g. psychiatrist, psychologist, general practitioner, social worker) for mental health problems in the past 12 months. Participants were asked to fill out the MHQoL, indicate whether the items cover the things that matter in their lives, and comment on the clarity of the individual items and the measure as a whole. Analysis of the provided comments confirmed the completeness and clarity of the MHQoL; no changes to the wording and sequence of items were deemed necessary.

### The Mental Health Quality of Life questionnaire (MHQoL)

The development process resulted in the Mental Health Quality of Life questionnaire (MHQoL). The MHQoL is a standardized, self-administered measure of quality of life that has been developed for use in people with subclinical and clinical mental health problems and across all types of mental health services. The MHQoL consists of two parts: a descriptive system, the MHQoL-7D and a visual analogue scale, the MHQoL-VAS. The MHQoL-7D comprises seven questions, covering seven dimensions (self-image, independence, mood, relationships, daily activities, physical health, future), each with four response levels (e.g. ranging from very satisfied (score = 3) to very dissatisfied (score = 0)). The MHQoL-7D sum score can vary from 0 to 21, with higher scores indicating better quality of life. The MHQoL-VAS records the self-esteemed general psychological well-being of the respondent on a horizontal scale ranging from zero ("worst imaginable psychological well-being") to ten ("best imaginable psychological well-being"). The MHQoL was developed in Dutch. The English version of the MHQoL is included in the supplemental material.

### Evaluation of psychometric properties

#### Study design and population

In order to evaluate the psychometric properties of the MHQoL, a web-based study was carried out. The study population consisted of 479 adult (18 years and older) mental healthcare service users and 110 adult members of the general population. During September 2018, participants were drawn from a consumer panel through a market research company (Dynata). The subsample of mental healthcare service users (aged 18 years or older) was selected from the larger panel based on the fact that respondents themselves indicated that they visited any health professional (e.g. psychiatrist, psychologist, general practitioner, social worker) for mental health problems in the past 12 months. The general population subsample was selected to represent the Dutch population in 2018 in terms of the distribution of age, sex, and education as recorded by Statistics Netherlands (Centraal Bureau voor de Statistiek). Participants received a financial incentive of €1.50 for their participation in the study.

#### Measures

In addition to the MHQoL, participants completed the self-report measures listed below.

The *five-level EuroQol five-dimensional questionnaire (EQ-5D-5L)* [19] is a five-item generic, preference-based self-report measure to describe and value health related quality of life (HRQoL). The EQ-5D-5L includes five dimensions (mobility, self-care, usual activities, pain/discomfort, and anxiety/depression) and a visual analogue scale (EQ-VAS) for overall health. Each dimension is divided into five response options describing the state per dimension (no problems, some problems, moderate problems, severe problems, and extreme problems/unable to). An index summary score can be generated by applying societal preference weights to the health state classification (scoring on the five dimensions) as completed by the respondent. Based on the Dutch tariff, total scores can range from − 0.446 to 1 [20], with higher values indicating better HrQoL as perceived by the general population. The EQ-VAS is a vertical scale ranging from zero ("worst imaginable health state") to 100 ("best imaginable health state") on which the respondents are asked to rate their overall health.

The *Manchester Short Assessment of quality of life* (MANSA) [13] is a 16-item self-report measure to assess quality of life in people with mental health problems. The MANSA is a shortened version of the Lancashire Quality of Life Profile (LQLP) [21] and consists of four dichotomous (yes/no) items covering objective quality of life aspects and 12 items assessing the satisfaction with life as a whole, job, financial situation, friendships, leisure activities, accommodation, personal safety, people that the person lives with, family and health. Each of the 12 satisfaction items is rated on a seven-point scale ranging from one (“couldn’t be worse”) to seven (“couldn’t be better)”. Summary scores can range from 12 to 84, with higher scores indicating better quality of life.

The *ICEpop CAPability measure for Adults* (ICECAP-A) [22] is a five-item generic, preference-based self-report measure of capability well-being for use in the adult population. The items cover five dimensions (stability, attachment, autonomy, achievement, and enjoyment), and each item has four response levels (e.g. none, a little, a lot and all). Index summary scores can range from 0 (representing the absence of capability) to 1 (representing full capability) [23].

The *Brief Symptom Inventory* (BSI) [24] is a 53-item self-report measure of psychopathology. The BSI is a shortened version of the Symptom Checklist-90 (SCL-90) [25] and covers nine dimensions (somatization, obsessive–compulsive, interpersonal sensitivity, depression, anxiety, hostility, phobic anxiety, paranoid ideation, and psychoticism). Each item is rated on a five-point scale ranging from zero (“not at all”) to four (“extremely”). The summary scale index of the BSI, the "Global Severity Index" (GSI), can range from 0 to 212, with higher scores indicating greater psychological distress.

#### Procedures

After providing digital informed consent, participants were asked to complete a web-based survey containing the MHQoL and questions about their socio-demographics (gender, date of birth, level of education, employment/activity) and mental health status (mental health problem, severity of mental health problem, duration of mental health problem). In addition, participants completed the EQ-5D-5L, MANSA, ICECAP-A, and BSI in order to evaluate convergent validity. After one week, the MHQoL was readministered to a randomly selected subset of 33% of participants reporting no change in their mental health related quality of life status after one week to assess test–retest reliability.

### Statistical analysis

Data on the demographic and clinical characteristics of the study sample were analysed using descriptive statistics. Internal consistency was assessed by item-total correlations and Cronbach's alpha coefficient in the total sample and subsample of mental healthcare service users. Cronbach's alpha values of 0.70–0.79 were considered acceptable, 0.80–0.89 good, and ≥ 0.90 excellent [26]. Test–retest reliability was assessed by intraclass correlation coefficient (ICC) using the two-way mixed effects, absolute agreement, single measurement model. Intraclass correlation coefficients of < 0.49, 0.5–0.74, 0.75–0.89, > 0.90 were considered poor, moderate, good, and excellent, respectively [27]. In order to assess convergent validity, Spearman's rank correlations were calculated between total MHQoL-7D scores and EQ-5D-5L sum score, EQ-5D-5L index, EQ-VAS, MANSA, ICECAP-A sum score, ICECAP-A index and BSI scores. Spearman's rank correlations of 0.10–0.29 were considered weak, 0.30–0.49 moderate, and ≥ 0.50 strong [28]. MHQoL-7D scores were expected to have a strong positive correlation with quality of life (EQ-5D, MANSA) and well-being (ICECAP-A) scores. Since quality of was demonstrated to be sensitive to variations in psychopathology (e.g. [29, 30]), the MHQoL was hypothesized to have a moderate negative correlation with the BSI. Within the subsample of mental healthcare service users, known group validity was assessed by evaluating the ability of the MHQoL-7D to detect significant group differences between participants by clinical status (clinical, BSI score ≥ 0.67 vs. non-clinical, BSI score < 0.67 [31]) and self-reported severity of mental health problems (severe vs. mild/moderate). The four original severity categories of mental health problems (mild, moderate, severe, very severe) were collapsed into two categories of (mild/moderate and severe). In addition, known-group validity was assessed in the total sample by testing whether the MHQoL-7D was able to discriminate between mental healthcare users and members from the general population. Group differences were examined using the Mann–Whitney *U* test. Mean MHQoL-7D group scores were expected to be significantly higher (i.e. better) in the group with non-clinical psychopathology, in the group with mild/moderate mental health problems, and in the group of members from the general population. All analyses were carried out using the Statistical Package for the Social Sciences (SPSS) version 24.0 (SPSS Inc., IBM Corporation, Armonk, New York, USA). Significance levels were set at *P* < 0.05 (two-tailed).

## Results

### Participant’s characteristics

Demographic and clinical characteristics of the study sample are presented in Table 1. The study sample comprised 479 mental healthcare service users and 110 members of the general population. The mean age of the total sample was 46.5 years (SD = 15.8), 341 (57.9%) were female, and most of the participants attained middle education (45.8%). In the subsample of mental healthcare service users, the most commonly reported mental health problems were depression (64.5%), dysthymia (41.8%), and anxiety disorder (42.0%). In the subsample of mental healthcare service users, the mental health problems were, as classified by the own perception of participants, in most cases of moderate severity (48.4%). The mean total MHQoL-7D and MHQoL-VAS scores were lower in the subsample of mental healthcare service users (11.5 (4.0) and 5.7 (2.0), respectively) than in the subsample of members of the general population (15.5 (2.9) and 7.5 (1.5), respectively).

**Table 1:** Demographic and clinical characteristics of study sample

	Total sample	Mental healthcare service users^a^	Members of the general population^a^
*N*	589	479	110
Age, years			
Mean (SD)	46.5 (15.8)	46.0 (15.7)	48.6 (16.1)
Range	18.1–85.8	18.1–85.8	18.9–80.7
Sex (N, %)			
Male	245 (41.6)	189 (39.5)	56 (50.9)
Female	341 (57.9)	287 (59.9)	54 (49.1)
Transgender	3 (0.5)	3 (0.6)	0 (0.0)
Education (*N*, %)^b^			
Lower education	135 (22.9)	113 (23.6)	22 (20.0)
Middle education	270 (45.8)	215 (44.9)	55 (50.0)
Higher education	184 (31.2)	151 (31.5)	33 (30.0)
Visited health professional for mental health problems in past 12 months (*N*, %)			
Yes	499 (84.7)	479 (100)	20 (18.2)
No	90 (15.3)	0 (0)	90 (81.8)
Type of health professional visited for mental health problems in past 12 months (*N*, %)^c^			
General practitioner	326 (55.3)	315 (65.8)	11 (10.0)
General nurse Practitioner mental healthcare	136 (23.1)	132 (27.6)	4 (3.6)
Social worker	61 (10.4)	59 (12.3)	2 (1.8)
Occupational physician	43 (7.3)	40 (8.4)	3 (2.7)
Psychotherapist	59 (10.0)	58 (12.1)	1 (0.9)
Psychologist	213 (36.2)	208 (43.4)	5 (4.5)
Psychiatrist	137 (23.3)	134 (28.0)	3 (2.7)
Other^d^	31 (5.3)	29 (6.1)	2 (1.8)
Mental health problem (*N*, %)^e^			
Depression	313 (53.1)	309 (64.5)	4 (3.6)
Dysthymia	208 (35.3)	200 (41.8)	8 (7.3)
Anxiety disorder	208 (35.3)	201 (42.0)	7 (6.4)
Personality disorder	105 (17.8)	99 (20.7)	6 (5.5)
Trauma- or stressor-related disorder	79 (13.4)	77 (16.1)	2 (1.8)
Autism or ADHD	77 (13.1)	76 (15.9)	1 (0.9)
Eating disorder	65 (11.0)	62 (12.9)	3 (2.7)
Obsessive-dompulsive disorder	46 (7.8)	43 (9.0)	3 (2.7)
Substance use disorder	36 (6.1)	35 (7.3)	1 (0.9)
Schizophrenia/psychosis	21 (3.6)	21 (4.4)	0 (0.0)
Other	19 (3.2)	16 (3.3)	3 (2.7)
Severity of current problems (*N*, %)^f^			
Mild	72 (12.2)	65 (13.6)	7 (6.4)
Moderate	241 (40.9)	232 (48.4)	9 (8.2)
Severe	139 (23.6)	137 (28.6)	2 (1.8)
Very severe	32 (5.4)	31 (6.5)	1 (0.9)
No problems anymore	15 (2.5)	14 (2.9)	1 (0.9)
No problems	90 (15.3)	0 (0.0)	90 (81.8)
MHQoL-7D			
Mean (SD)	12.3 (4.1)	11.5 (4.0)	15.5 (2.9)
Range	0–21	0–21	8–21
MHQoL-VAS			
Mean (SD)	6.0 (2.0)	5.7 (2.0)	7.5 (1.5)
Range	0–10	0–10	2–10

*ADHD* Attention-Deficit/Hyperactivity Disorder, *MHQoL* Mental Health Quality of Life questionnaire, *SD* Standard Deviation, *VAS* Visual Analogue Scale

^**a**^Part of total sample

^b^Lower, middle, and higher education refers to ISCED [32] 2011 levels 0–2 (early childhood education, primary education, lower secondary education), 3–4 (upper secondary education, post-secondary non-tertiary education), and 5–8 (short-cycle tertiary education, bachelor or equivalent, master or equivalent, doctoral or equivalent), respectively.

^c^Some participants indicated that they visited more than one health professional for their mental health problems in the past 12 months.

^d^For example community psychiatric nurse, hypnotherapist, vitality coach.

^e^Some participants indicated to have > 1 mental health problem (mean number of mental health problems in total population was 2.4 (SD = 1.4)).

^f^Severity was classified based on the own perception of participants

### Reliability

Table 2 presents the internal consistency reliability and test–retest reliability coefficients for the individual MHQoL-7D items. In the total sample, the Cronbach’s alpha coefficient for the total MHQoL-7D was 0.85 and item-total correlations ranged from 0.48 to 0.71. None of the items could be deleted without a decrease of Cronbach's alpha. Test–retest reliability, as assessed by ICC, was 0.85 for the total MHQoL-7D. ICCs for individual items ranged from 0.51 to 0.77.

**Table 2:** Item-total correlations, alpha if item deleted and intraclass correlation coefficients for individual MHQoL-7D items

	Total sample		Mental healthcare service users		Test–retest reliability subsample (*N* = 195)
Item	Item-total correlation^a^	α if item deleted^a^	Item-total correlation^a^	α if item deleted^a^	ICC
1—Self-image	0.69	0.81	0.67	0.79	0.73
2—Independence	0.57	0.83	0.54	0.81	0.60
3—Mood	0.69	0.81	0.65	0.79	0.70
4—Relationships	0.49	0.84	0.44	0.83	0.70
5—Daily activities	0.63	0.82	0.60	0.80	0.51
6—Physical health	0.48	0.84	0.45	0.83	0.77
7—Future	0.71	0.81	0.69	0.79	0.76

*ICC* Intraclass Correlation Coefficient

^a^Time point = baseline

^b^All significant at *P* < 0.001 (2-tailed)

### Convergent validity

Spearman's rank-order correlations between MHQoL-7D scores and total scores of convergent measures are presented in Table 3. As hypothesised, the MHQoL showed strong positive correlations with the EQ-5D-5L, MANSA, and ICECAP-A scores. Moreover, there was a strong negative correlation between increasing MHQoL-7D scores and psychopathology scores as measured by the BSI.

**Table 3:** Spearman's rank-order correlations between MHQoL-7D scores and total scores of convergent measures^a,b^

Measure	Total sample	Mental healthcare service users	Members of the general population
EQ-5D-5L sum score	− 0.58	− 0.53	− 0.47
EQ-5D-5L index	0.63	0.59	0.49
EQ-VAS	0.65	0.61	0.54
MANSA	0.75	0.71	0.69
ICECAP-A sum score	0.71	0.65	0.62
ICECAP-A index	0.71	0.66	0.62
BSI	− 0.64	− 0.57	− 0.65

*BSI* Brief Symptom Inventory, *EQ-5D-5L* five-level EuroQol five-dimensional questionnaire, *ICECAP-A* ICEpop CAPability measure for Adults, *MANSA* Manchester Short Assessment of quality of life, *VAS* Visual Analogue Scale

^a^Time point = baseline

^b^All significant at *P* < 0.001 (2-tailed)

### Known-group validity

A Mann–Whitney *U* test indicated that MHQoL-7D scores were significantly higher in participants with non-clinical psychopathology (Mdn = 15) than in participants with clinical psychopathology (Mdn = 11) (*U* = 11,256; *P* < 0.001; *r* = 0.39). In addition, MHQoL-7D scores were significantly higher in participants with mild/moderate mental health problems (Mdn = 13) than in participants with severe mental health problems (Mdn = 9) (*U* = 12,300; *P* < 0.001; *r* = 0.42), and in members from the general population (Mdn = 16) than in mental healthcare users (Mdn = 12) (*U* = 7.698; *P* < 0.001; *r* = 0.47).

## Discussion

This paper reports on the development and psychometric evaluation of new quality of life measure for use in people with mental health problems—the Mental Health Quality of Life questionnaire (MHQoL). The MHQoL was designed to comprehensively provide information about the quality of life dimensions known to be relevant across and valued highly by people with mental health problems. Overall, the results of the present study suggest that the Dutch version of the MHQoL is a psychometrically sound measure of quality of life in Dutch people with mental health problems.

The face and content validity of the Dutch version of the MHQoL in Dutch people with mental health problems are supported by a multi-source, service user-oriented development process. Evaluation of the face and content validity by a focus group and online qualitative study confirmed the completeness and clarity of the MHQoL in this context. In addition, in the current study, the MHQoL demonstrated good internal consistency and good test–retest reliability over a 1-week interval. Moreover, correlations between the Dutch version of the MHQoL and related measures supported convergent validity. As expected, higher scores on the MHQoL were strongly associated with higher scores on the ICECAP-A, EQ-5D-5L and MANSA. The MHQoL was more strongly associated with the ICECAP-A and MANSA than with the EQ-5D-5L. This is expected since the ICECAP-A and MANSA cover more dimensions included in the MHQoL compared to the EQ-5D-5L. In addition, there was a strong negative correlation between MHQoL scores and severity of mental health problems as measured by the BSI. Although quality of life has been found to be sensitive to variations in psychopathology (e.g. [29, 30]), it is remarkable that the strength of the correlation between the MHQoL and BSI is comparable to the correlations between the MHQoL and other quality of life (EQ-5D-5L, MANSA) and well-being (ICECAP-A) measures. This finding raises the question what the differences between and interrelationships among quality of life, well-being and psychopathology are, also in terms of the underlying constructs. This is an interesting and important question, but one that falls beyond the scope of the current study and requires attention in future research. Known-group validity was supported by the ability of the MHQoL to detect significant differences in overall MHQoL levels between service users and the general population, between those reporting severe mental health problems and mild/moderate mental health problems, and between those with clinical psychopathology and with non-clinical psychopathology.

The MHQoL offers several important advantages over most existing quality of life measures. The MHQoL was designed based on a comprehensive overview of the quality of life dimensions most relevant to people with mental health problems [15, 16]. Hence, the MHQoL is likely to be more sensitive to the benefits of mental healthcare interventions than generic quality of life measures. At the same time, it needs noting that this likely increase in sensitivity within the mental health domain may compromise the comparability of outcomes across sectors. However, in contrast to existing disease-specific quality of life measures, the MHQoL does still allow comparisons to be made across conditions *within* the mental health field. In addition, the MHQoL is relatively short and easy to complete by respondents in comparison to available quality of life measures used in people with mental health problems (average number of items = 35 [12]). The favourable ease of use of the MHQoL may support the use of the MHQoL in clinical and research settings alongside more clinically oriented measures, and would thereby accommodate the growing interest in measuring and monitoring the impact of mental health(care) on peoples’ lives [2]. Although collecting ‘traditional’ outcomes, such as data on symptom remission, will remain essential, complementing it with outcome data on quality of life will offer a more complete understanding of the effectiveness of mental healthcare services, also from the perspective of those suffering from mental health problems. Moreover, the MHQoL can facilitate economic evaluations of mental health services, as further highlighted below.

The growing interest in comprehensive and sensitive outcome measures that can be used broadly in the mental health domain, may be underscored by the fact that recently more measures than only the MHQoL have been developed and introduced. To our knowledge, the only published examples of recently developed quality of life measures that cover all dimensions valued highly by people with mental health problems are the Recovering Quality of Life (ReQoL) measures [18]. Although the MHQoL and the ReQoL measures share the same goal, target population and theoretical basis (i.e. dimensions), they differ in a number of important ways, including the operationalization of their dimensions, the number of items (7 (MHQoL) vs. 10 (ReQoL-10) and 20 (ReQoL-20)), the recall period (“Today” (MHQoL) vs. “Last week” (ReQoL)), and the integration of the physical dimension in the measure (integrated (MHQoL) vs. supplemental (ReQoL)). The psychometric properties in terms of feasibility, reliability, validity and responsiveness of both ReQoL measures were reported to be satisfactory [17]. A direct comparison of the psychometric performance of the MHQoL and de ReQoL measures based on the published findings could not be performed because of differences in sampling and measurement methods between the studies. Hence, we encourage future research to explore how the measures relate to one another and, for instance, which measure is preferred to be used in which context.

Several limitations to this study need to be acknowledged. First, as the presented study is a first psychometric evaluation of the MHQoL, future studies are needed to replicate and extend the findings from this initial evaluation. As the MHQoL was designed to adequately capture mental health-related quality of life and through that the benefits of mental healthcare interventions, in future studies special attention should be given to the evaluation of the sensitivity to change. In addition, future research is required to compare the sensitivity of the MHQoL to other (generic) quality of life measures and establish the effect of the use of a weighted sum score on the psychometric properties of the MHQoL. Second, the findings of the present study might have been subject to selection bias as participants were recruited by a market research company. Although people who voluntarily take part in online studies might differ from the general (patient) population, the sampling methodology resulted in a heterogeneous sample in terms of age, sex and education. Other consequences of the sampling procedure are that the rate of non-participation could not be determined, a relatively limited number of people with severe mental health problems participated, and a comprehensive psychiatric assessment by a mental health professional could not be performed, and hence, clinical and research diagnoses are missing. Future studies are needed to evaluate the psychometric properties in a clinically heterogeneous sample of mental healthcare service users. Third, in order to avoid subjective weighting of health states experienced over longer periods of time, and in line with other generic quality of life measures such as the EQ-5D, the recall period was set to "today". Recent research on issues related to different recall-periods and fluctuating health states indicates [33] that this choice may be influential and needs consideration also in the practical application of a measure. A main limitation of the here chosen recall period may be that fluctuations in quality of life may be missed and that obtained observations could be biased. This potential bias could, however, be reduced by administrating measures with a shorter recall period on a specific date, on a day with problems as well as on day without problems or by a more frequent administration of such measures [22]. In addition, this form of bias could be reduced by complementing the administration of the measure by diary completion in order to be able to assess whether the measure was administered on a day with or without problems. Fourth, in the present study, only the original Dutch version of the MHQoL was evaluated. English and German translations have been produced but are not yet tested for their psychometric properties. Broader validations of translated versions of the MHQoL in other countries are encouraged, in which cultural differences in relation to mental health should also be considered. Fifth, as we tested the MHQoL in a sample of people aged 18 years and older, the MHQoL cannot be recommended for use in people younger than 18 without further psychometric evaluation, although, given the phrasing and domains, it may be considered potentially suitable for adolescents as well. Recommendations for future research include further psychometric testing, also in an international context, the development of a preference-based scoring algorithm to make the MHQoL suitable for use in cost-utility studies, and the direct comparison of the MHQoL with other recently developed quality of life measures for use in the mental health field such as the ReQoL measures. In addition, in order to increase the clinical relevance of the MHQoL, norm scores should be established to aid the interpretation of the MHQoL.

Notwithstanding these limitations, this study indicates that the MHQoL is a psychometrically sound measure in the Dutch context and, therefore, holds a promising capability as a simple, short and effective measure to assess quality of life in people with mental health problems. In order to make the MHQoL suitable for use in cost-utility analyses of mental healthcare interventions, preference weights will be estimated by use of a discrete choice experiment [34] in due course. By doing so, the MHQoL may facilitate sound economic evaluations of mental health interventions.

## Availability of the MHQoL

The MHQoL, its scoring manual and user conditions can be found at https://www.imta.nl/mhqol/. Erasmus School of Health Policy & Management, Erasmus University Rotterdam, Rotterdam, The Netherlands, is the copyright holder of the Mental Health Quality of Life Questionnaire (MHQoL).

## Supplementary Information

Below is the link to the electronic supplementary material.

Supplementary file1 (PDF 71 kb)

## Data Availability

The data that support the findings of this study are available from the corresponding author (FK) upon reasonable request.

## Footnotes

1 The item labels of the MHQoL correspond as follows to the labels of the dimensions of the conceptual framework (item label MHQoL = dimension label): self-image = self-perception; independence = autonomy; mood = well-being and ill-being; relationships = relationships and belonging; daily activities = activity; physical health = physical health; future = hope and hopelessness.
