# Supplementary material for: The Mental Health Quality of Life Questionnaire (MHQoL): development and first psychometric evaluation of a new measure to assess quality of life in people with mental health problems
Source: Qual Life Res. 2021 Jul 9;31(2):633–43. doi: 10.1007/s11136-021-02935-w (PMC8847188; doi:10.1007/s11136-021-02935-w)
Supplement: Supplementary file 1 — Supplementary file1 (PDF 71 kb) [file 11136_2021_2935_MOESM1_ESM.pdf]

# MHQoL

Please indicate below which statements best describe your situation **TODAY** by ticking **ONE** box in each of the seven subjects.

**SELF-IMAGE**

I think very positively about myself ☐

I think positively about myself ☐

I think negatively about myself ☐

I think very negatively about myself ☐

**INDEPENDENCE** *For example: freedom of choice, financial, co-decision making*

I am very satisfied with my level of independence ☐

I am satisfied with my level of independence ☐

I am dissatisfied with my level of independence ☐

I am very dissatisfied with my level of independence ☐

**MOOD**

I do not feel anxious, gloomy, or depressed ☐

I feel a little anxious, gloomy, or depressed ☐

I feel anxious, gloomy, or depressed ☐

I feel very anxious, gloomy, or depressed ☐

**RELATIONSHIPS** *For example: partner, children, family, friends*

I am very satisfied with my relationships ☐

I am satisfied with my relationships ☐

I am dissatisfied with my relationships ☐

I am very dissatisfied with my relationships ☐

**DAILY ACTIVITIES** *For example: work, study, household, leisure activities*

I am very satisfied with my daily activities ☐

I am satisfied with my daily activities ☐

I am dissatisfied with my daily activities ☐

I am very dissatisfied with my daily activities ☐

*MHQoL (English) © Copyright Erasmus University Rotterdam, Erasmus School of Health Policy & Management 2018. All rights reserved.*

**PHYSICAL HEALTH**

- I have no physical health problems ☐
- I have some physical health problems ☐
- I have many physical health problems ☐
- I have a great many physical health problems ☐

**FUTURE**

- I am very optimistic about my future ☐
- I am optimistic about my future ☐
- I am gloomy about my future ☐
- I am very gloomy about my future ☐

**PSYCHOLOGICAL WELL-BEING**

On the scale below, please indicate with an X how you rate your psychological well-being. 0 represents the worst imaginable psychological well-being, while 10 represents the best imaginable psychological well-being.

Worst imaginable psychological well-being — Best imaginable psychological well-being

0 1 2 3 4 5 6 7 8 9 10

*MHQoL (English) © Copyright Erasmus University Rotterdam, Erasmus School of Health Policy & Management 2018. All rights reserved.*
